# Model Construction for Undergraduate Student College Adjustment

**DOI:** 10.3390/ijerph17197090

**Published:** 2020-09-28

**Authors:** Sona Lee, Hye Young Ahn

**Affiliations:** College of Nursing, Eulji University, Daejeon 34824, Korea; sonasona326@naver.com

**Keywords:** adjustment, child rearing, social support, ego, social networking, aggression

## Abstract

*Background:* College students are known to struggle with a number of difficulties, such as their future careers and interpersonal relationships, as well as job-seeking stress. This study aimed to develop and test a structural model for undergraduate student college adjustment. *Methods*: The data collection period ranged from November 2019 to January 2020. A questionnaire was distributed to a total of 300 college students; a total of 290 copies were ultimately used for analysis. *Result:* The model fit indexes of the final model were χ^2^ = 427.707 (*p* < 0.001), DF = 173, χ^2^/DF = 2.47, GFI = 0.88, Adjusted Goodness of Fit Index (AGFI) = 0.84, Comparative Fit Index (CFI) = 0.91, Incremental Fit Index (IFI) = 0.92, Standardized Root Mean-square Residual (SRMR) = 0.07, and Root Mean-Square Error of Approximation (RMSEA) = 0.07. All of the model fit indexes were acceptable, and seven of the thirteen paths in the final model were significant. The factors that influenced college adjustment were social support (r = 0.39) and ego-identity (β = 0.73), explaining 57.1% of the variance. *Conclusions:* To increase college adjustment, it is necessary to consider the relationship of adjustment with college students’ surrounding environments, such as their family, friends, and professors, and how students can improve their ego-identity.

## 1. Introduction

Currently, college students are struggling with problems such as their career paths, employment, and personal relationships. College students in Korea attend college after receiving a high school education focused on entrance exams with a set curriculum. As a result, they have a difficult time proactively learning and coping with interpersonal relationships and their major fields [1]. In addition, some students are unable to adjust to college due to poor interpersonal relationships and stress for the future. If the degree of maladjustment is high, students may consider stopping their studies [2].

According to the basic education statistics released by the Korean Ministry of Education, the rate of college entrance after graduation from high school was 69.7% in 2018 and 70.4% in 2019 [3]. On the other hand, the rate of discontinuation due to a leave of absence or withdrawal was 6.9% in 2018 and 6.8% in 2017 [3]. Although a large number of students attend college, many give up because they cannot adjust to college at a similar rate every year. Therefore, it is important to understand the environmental and personal disposition of college students and develop the ability to actively find and solve the causes of these problems.

Humans engage in developmental tasks through continuous stages throughout their lives and cope with changing environments. For college students, the transition from adolescence to adulthood corresponds to the period before the students enter their social life and must adjust to various factors such as their academics, careers, values, and friends [4]. Students also attend college to develop their own abilities to solve various problems that arise. In addition, students must learn effective communication so that they can think critically and have smooth interpersonal relationships; they must also be able to actively cope with the personal, psychological, and social problems encountered in college life [5]. As such, college is an important period in the developmental process of life, during which various roles are learned and acquired.

Despite the importance of adapting to the various environments of college, many college students experience stress due to academic and economic problems, career difficulties, and employment difficulties after graduation. These experiences cause psychological and emotional difficulties, affecting their lives and leading to maladjusted social interactions [4]. In the field of nursing, college adjustment is studied as the overall life adjustments of nursing students [6] and the cultural adjustment of international students [7]. However, studies on structural models of adjustment to college among general university students remain insufficient. Therefore, by understanding the various personal and relational aspects of college students, we sought to determine whether these factors have a positive or negative effect on college adjustment.

Bandura’s social cognitive theory believes that behavior is determined by interactions with individual characteristics and the environment [8]. Therefore, this study attempts to construct a hypothetical model by identifying the factors that influence college adjustment based on Bandura’s social cognitive theory. It also tries to identify the direct and indirect relationships and degree of variables that influence college adjustment. This study can be used as basic data to facilitate understanding of college adjustment and develop mediation plans and programs for positive adjustments to college life.

The purpose of this study is to establish a hypothetical model and verify the model’s suitability by determining the factors affecting college adjustments and the relationship between each factor. The specific purposes of this study are as follows:

- Build a hypothetical research model for college adjustments.
- Propose a model that explains college adjustments by verifying the model fit between the hypothetical model and the collected data.
- Identify the causal relationships among the variables that explain college adjustments.

### Theoretical Framework and Hypothetical Model

This study constructed a conceptual framework based on Bandura’s social cognitive theory. In Bandura’s theory, behavior is determined by an individual’s characteristics and interactions with the environment [8]. In this theory, the individual’s follow-up behavior is created through the interaction of the individual’s characteristics, the individual’s behavior, and the environment in which the individual is located. Understanding this interaction is the only way to understand the psychological functioning of humans [9]. In addition, in social cognition theory, direct learning has an important effect on behavior, but indirect experiences such as newspapers, TV, and movies can also be used [8]. Recently, because of the development of smart phones, college students mainly use social networking sites(SNSs). Thus, it is necessary to understand the use of SNSs.

The behavior that results from an individual’s actions determines what actions should be taken based on various environmental influences [9]. An individual observes the actions of others and then learns from and simulates the results [10]. Therefore, behavioral factors are influenced by personal factors and environmental factors, and personal factors are influenced by the environmental factors.

**Figure 1:**
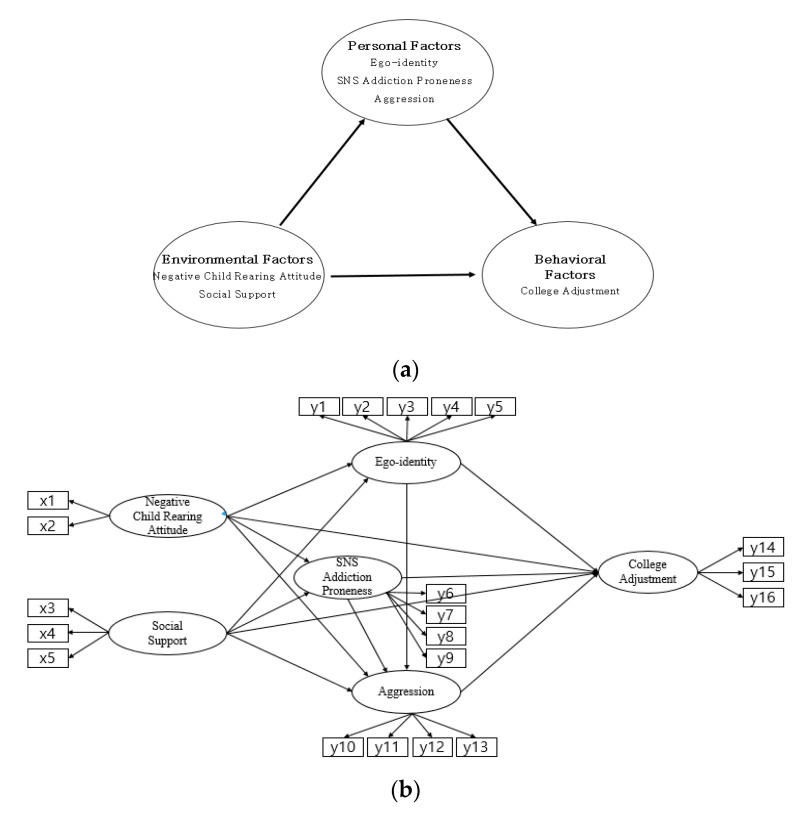
(**a**) Theoretical framework; (**b**) hypothetical model.

Based on this theoretical framework, a hypothetical model was constructed to identify the factors influencing college students’ adjustments to college. The hypothetical model is composed of the effects of negative parental child rearing attitudes; the direct effects of social support-based environmental factors on college adjustment; and the effects of behavioral factors, using personal factors such as ego-identity, SNS addiction proneness, and aggression as parameters. This hypothetical model has two exogenous potential variables (a negative parental child rearing attitude and social support) and four endogenous potential variables (ego-identity, SNS addiction proneness, aggression, and college adjustment) (Figure 1).

## 2. Materials and Methods

### 2.1. Study Participants

The subjects of this study were students enrolled in universities in S city, D city, C area, and J area selected through convenience sampling. We visited 7 universities and collected data in the classroom with permission from the professors and students of the department. The criteria for inclusion of research subjects are students who are enrolled in a 4-year university who have heard and agreed to the purpose of this study. Exclusion criteria are students on leave of absence and students who disagree with this study. When verifying a model via a structural equation, the minimum recommended sample size should be 200 or more [11]. In this study, a questionnaire was distributed to 300 college students enrolled in 7 universities while considering the dropout rate. Excluding 10 with insufficient responses, 290 questionnaires were used as the analysis data.

For gender, 172 subjects were female (59.3%), and 118 were male (40.7%). The first grade was 97 (33.4%), the second grade was 82 (28.3%), the third grade was 63 (21.7%), and the fourth grade was 48 (16.6%). In total, 78 (26.9%) students were in the health sciences, 61 (21.0%) students were in the engineering. Additionally, 171 (59.0%) had no religion, 75 (25.9%) were Christian. For educational background, 186 (64.1%) of the fathers and 161 of the mothers (55.5%) graduated from college. For economic status, 157 respondents (54.1%) answered ‘medium’. Satisfaction with one’s major was average for 109 students (37.6%). Most of participants (270 college students, 93.1%) used SNSs. A total of 92 respondents (31.7%) said that they used SNSs for an average of 120-179 minutes a day. In addition, 213 students (73.4%) accessed SNSs every day, and 159 respondents (54.8%) thought that they could have a wide range of relationships through SNSs. For SNS contacts, 106 participants (36.6%) noted that they had ‘a small number’ of contacts that were rarely seen offline and maintained mainly through a SNS, and 249 (85.9%) had experienced no problems with friends due to social media (Table 1).

### 2.2. Research Instruments

The printed questionnaire used in this study was composed of negative child rearing attitude 15 items, social support 12 items, ego-identity 50 items, SNS addiction proneness 24 items, Aggression 27 items, and college adjustment 12 items.

#### 2.2.1. Negative Child Rearing Attitude

In this study, negative child rearing attitude refers to caregivers who do not accept their children as they are, have a rejecting attitude, and plan everything for their children while trying to control them excessively [12]. We applied rejection and overprotective parenting attitudes in the scale [13] that Jo (2011) adapted from the shortened EMBU (Egna Minnen Betraffande Uppfostran) [14] by Arrindell et al. (2001). The number of items was 7 for rejection and 8 for over protection. A 4-point Likert scale was used for the questionnaire, and scores ranged from 1 (for ‘never’) to 4 for ‘most of the time’. The higher the score was, the higher the negative child rearing attitude. Cronbach’s α for each sub-domain was 0.75 for maternal rejection and 0.72 for maternal overprotection in Jo’s study [13] and 0.81 and 0.78 in this study.

#### 2.2.2. Social Support

In this study, social support is considered a positive type of support that an individual can obtain via interpersonal relationships, which mitigates the negative effects of stressful situations [15]. We used the scale that Shin and Lee (1999) [16] adapted from the Multidimensional Scale of Perceived Social Support (MSPSS) by Zimet et al. (1998) [17]. This scale consists of 4 items for family support, 4 for support from friends, and 4 for support by a significant other, totaling 12 items. Each item used a 5-point Likert scale, ranging from 1 for ‘not at all’ to 5 for ‘very much’. The higher the sum of the scores, the higher the social support. In a past study by Shin and Lee [16], all items were translated for use with Korean college students, and the total Cronbach’s α was 0.89. In addition, in a study on middle-aged women using this scale [18], the total Cronbach’s α was 0.90, and 0.83 for the sub-domains; family support was 0.83, friend support was 0.93, and significant other support was 0.87. In this study, the Cronbach’s α values of the sub-domains were 0.88, 0.92, and 0.94, and the Cronbach’s α of the total social support was 0.92.

#### 2.2.3. Ego-Identity

In this study, the sense of ego-identity refers to the feeling of an individual who recognizes him- or herself through his or her ideals, behaviors, and social roles [19]. Here, we used the Korean scale of self-identity developed by Park (2003) in 1996 [20] and reconstructed in 2003 [21]. This study used 10 items for initiative, 10 items for self-receptiveness, 10 items for confirmation for the future, 10 items for goal orientation, and 10 items of intimacy. The 5-point Likert scale ranges from 1 for ‘it does not apply at all’ to 5 for ‘it applies very much’. The higher the sum of the scores, the higher the sense of self-identity. In Park’s study [21], Cronbach’s α was 0.94 in total, 0.74 for initiative, 0.83 for self-receptiveness, 0.83 for confirmation for the future, 0.77 for goal orientation, and 0.74 for intimacy. In this study, the total was 0.96, and the sub-domains were 0.84, 0.88, 0.88, 0.85, and 0.85, respectively.

#### 2.2.4. SNS Addiction Proneness

In this study, the proneness of addiction to SNSs refers to withdrawal symptoms and obsession with SNS use and excessive communication and immersion, making daily life difficult and making a student unable to control his or her own behavior [22]. We used the SNS addiction proneness scale [23] for college students developed by Jung and Kim (2014). This scale consists of 7 items for the disturbance of adaptive life and control, 7 items for preoccupation and tolerance, 5 items for the avoidance of negative emotions, and 5 items for virtual life orientation and withdrawal. The 4-point Likert scale ranges from 1 point for ‘not at all’ to 4 points for ‘very much’. The higher the sum of the scores, the higher the smartphone addiction. In Jung and Kim’s study [23], Cronbach’s α was 0.92 in total, with 0.84 for disturbance of adaptive life and control, 0.80 for preoccupation and tolerance, 0.81 for avoidance of negative emotions, and 0.77 for virtual life orientation and withdrawal. In this study, the total was 0.94, and the sub-domains were 0.86, 0.83, 0.85, and 0.85, respectively.

#### 2.2.5. Aggression

Aggression means having the direct intention to inflict pain or damage to others [24]. We used a scale [25] that Seo and Kwon (2002) adapted and developed based on the Aggression Questionnaire (AQ) scale [26] of Buss and Perry (1992) (Korean version). This scale is composed of 9 items for physical aggression, 5 items for verbal aggression, 5 items for anger, and 8 items for hostility. The 5-point Likert scale ranges from 1 point for ‘not at all’ to 5 points for ‘very much’; the higher the sum of the points, the higher the aggressiveness. Cronbach’s α was 0.89 overall, with 0.74 for physical aggression, 0.73 for verbal aggression, 0.67 for anger, and 0.76 for hostility in Seo and Kwon’s study [25]. In this study, the total was 0.92, followed by the sub-domains with 0.82, 0.76, 0.72, and 0.83, respectively.

#### 2.2.6. College Adjustment

College adjustment is the process through which college students learn to lead a successful college life and feel a high degree of satisfaction through their active interactions with members and environments within the college, allowing them to actively cope with the academic, career, and interpersonal problems that emerge in college life [27]. We used the college adjustment scale [27] developed by Jeong and Park (2009). In this study, 4 items for academic activities, 4 items for career preparation, and 4 items for personal psychology were used. On a 5-point Likert scale, the score ranged from 1 point for ‘very much’ to 5 points for ‘not at all’. The higher the sum of the scores, the greater the college adjustment. Cronbach’s α was 0.86 overall, with 0.78 for academic activities, 0.77 for career preparation, and 0.77 for personal psychology in Jeong and Park’s study [27]. In this study, the total was 0.88, followed by the sub-domains with 0.76, 0.81, and 0.81, respectively.

### 2.3. Data Collection and Ethical Considerations

This study was approved by the E University Institutional Review Board (EU19-94) before data collection. Data collection was conducted from 29 November 2019 to 20 January 2020. We met with the professors and student councils from the department by phone. In addition, the purpose and necessity of the study and the research methods were explained, and informed permission was obtained. After that, the researcher visited the college and entered the lecture room to provide information on the research to 300 college students. The purpose, necessity, and data collection procedures for this study were explained, and after obtaining consent for cooperation, the study was initiated with voluntary participation. We explained that the collected questionnaire would be confidential and used only for research and that there will be no penalty for quitting during participation. The questionnaire was distributed at the site, filled out by the subjects, sealed in an envelope, and collected by the researcher. The time required for the questionnaire response was about 20 min.

### 2.4. Data Analysis

This paper is a model construction study that seeks to identify and explain the factors affecting college adjustment and verify the validity of the model and the relationships between its variables.

The collected data were analyzed using SPSS for Windows 25.0 and AMOS 25.0. The general characteristics of the subjects were analyzed by descriptive statistics, and the reliability of the scales was verified by Cronbach’s alpha. The sample normality was verified using skewness and kurtosis. The multicollinearity between the measured variables was confirmed by Pearson’s correlation coefficient, tolerance, and the variation inflation factor (VIF).

To test the fit of the model, χ^2^ statistic, χ^2^ standard statistic (χ^2^/DF), Goodness of Fit Index (GIF), Adjusted Goodness of Fit Index (AGFI), Comparative Fit Index (CFI), Incremental Fit Index (IFI), Standardized Root Mean-square Residual (SRMR), and Root Mean-Square Error of Approximation (RMSEA) were used. The parameter estimates for the measurement variables were analyzed as the Standardized Regression Weight (SRW), Regression Weight (RW), Critical Ratio (C.R.), Squared Multiple Correlations (SMC), and Standard Error (SE). The statistical significance of the direct effect, indirect effect, and total effect was confirmed by bootstrapping.

## 3. Results

### 3.1. Descriptive Statistics and Confirmatory Factor Analysis of the Measured Variables

For negative child rearing attitude, the average maternal overprotection was 1.88 points (1–4 points), and maternal rejection was 1.39 points (1–4 points) on average. For social support, familial support and friend support averaged 4.17 points (1–5 points). For self-identity, self-receptiveness averaged 3.75 points (1–5 points), preoccupation and tolerance averaged 1.96 points in SNS addiction tendency (1–4 points), and anger averaged 2.28 points in aggression (1–5 points). In terms of college adjustment, personal psychology and academic activities averaged 3.59 points (1–5 points) (Table 2).

In a structural equation model, skewness and kurtosis are analyzed to confirm the normality of the sample. If the absolute value of skewness is greater than 3, and the absolute value of kurtosis is greater than 7, then there is a problem in assuming a normal distribution [12]. In this study, the skewness was −1.06–1.78, and the absolute value of the skewness was less than 3. Kurtosis is −0.63~3.66, and the absolute value is less than 7. Therefore, the data in this study met the criteria and confirmed the normality of all questions. In addition, if the absolute value of the correlation coefficient between the measured variables is 0.8 or more, tolerance is 0.1 or less, and the Variation Inflation Factor (VIF) is 10 or more, multicollinearity exists [28]. The correlation coefficient between the measured variables was found to be −0.45–0.76, the tolerance limit was 0.55–0.82, and the variance expansion coefficient was 1.22–1.83, so there was no multicollinearity.

The standardization factor of the scales used for the confirmatory factor analysis satisfies the concept validity of 0.52–0.92. Concentrated validity is satisfied if the variance extraction index (AVE) is 0.5 or more, and the conceptual reliability (CR) is 0.7 or more [12]. In general, the higher the internal consistency (which indicates the reliability of the tool), the higher the validity [29]. However, if an exception to this rule is made, it is more desirable to sacrifice internal consistency because the content validity of the concept being measured by the scale is the most important factor [30]. Even if the values of AVE and CR (which indicate the internal consistency of the confirmatory factor analysis) do not meet the criteria, if the scale contains a representative element of the characteristics to be measured, it is possible to make a wider inference under various conditions and situations. Therefore, this method was used for the present research without deleting the variable.

Discriminant validity checks whether the AVE value is greater than the squared value of the correlation [30]. In this study, the AVE of the latent variables was 0.38–0.63, and the correlation coefficient between the latent variables was −0.32–0.73. Thus, the discriminant validity of the question composition concept was satisfied because AVE was larger than the square of the correlation coefficient.

### 3.2. Test of the Model Fit of the Hypothetical Model

When evaluating the model, a model fit is judged to be good if the fit is less than χ^2^/DF 3 and has a GFI of 0.90 or more, an AGFI of 0.80 or more, a CFI and IFI of 0.90 or more, an SRMR of 0.08 or less, and an RMSEA of 0.05 to 0.08 [31,32,33]. After analyzing the model fit index of the hypothetical model in this study, the following results were found: χ^2^ = 427.707 (*p* < 0.001), DF = 173, χ^2^/DF = 2.47, GFI = 0.88, AGFI = 0.84, CFI = 0.91, IFI = 0.92, SRMR = 0.07, and RMSEA = 0.07. Thus, this model offers an acceptable level for all fit indices.

### 3.3. Analysis of the Effects of the Hypothetical Model

**Figure 2:**
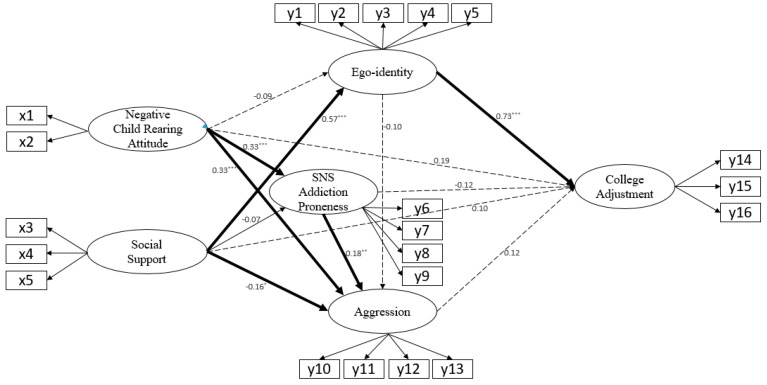
Path diagram of the modified model. *** *p* < 0.001, ** *p* < 0.01, * *p* < 0.05, X1 = maternal rejection; X2 = maternal overprotection; X3 = family support; X4 = friend support; X5 = significant other support; Y1 = initiative; Y2 = self-receptiveness; Y3 = confirmation for the future; Y4 = goal orientation; Y5 = intimacy; Y6 = disturbance of adaptive life and control; Y7 = preoccupation and tolerance; Y8 = avoidance of negative emotions; Y9 = virtual life orientation and withdrawal; Y10 = physical aggression; Y11 = verbal aggression; Y12 = anger; Y13 = hostility; Y14 = academic activities; Y15 = career preparation; Y16 = personal psychology.

Of the total 13 paths, 7 paths were statistically significant. Ego-identity was significantly affected by social support (r = 0.57, *p* < 0.001), and the explanatory power for ego-identity was 35.7%. SNS addiction proneness was statistically significantly affected by negative child rearing attitudes (r = 0.33, *p* < 0.001), and the explanatory power of SNS addiction proneness was 12.7%. Aggression was statistically significantly affected by negative child rearing attitudes (r = 0.33, *p* < 0.001), social support (r = −0.16, *p* = 0.037), and SNS addiction proneness (r = 0.18, *p* = 0.004), and the explanatory power of aggression was 29.4%. College adjustment was statistically significantly affected by ego-identity (r = 0.73, *p* < 0.001), and the explanatory power for college adjustment was 57.1% (Table 3, Figure 2).

The effect analysis for the hypothetical model is shown in Table 3 for direct effects, indirect effects, and total effects. Social support for ego-identity was significant in its direct effects (r = 0.567, *p* = 0.003) and total effects (r = 0.567, *p* = 0.003). The direct effects (r = 0.331, *p* = 0.005) and total effects (r = 0.331, *p* = 0.005) were significant for negative child rearing attitudes on SNS addiction proneness. For the effects of negative child rearing parenting attitudes on aggression, the direct effects (r = 0.328, *p* = 0.005), indirect effects (r = 0.069, *p* = 0.005), and total effects (r = 0.396, *p* = 0.003) were all significant. In social support for aggression, the indirect effects (r = −0.067, *p* = 0.116) were not significant, but the direct effects (r = −0.161, *p* = 0.048) and total effects (r = −0.228, *p* = 0.005) were significant. The direct effects (r = 0.182, *p* = 0.017) and the total effects (r = 0.182, *p* = 0.017) were significant for SNS addiction proneness on aggression. The direct effects (r = 0.098, *p* = 0.385) were not significant for social support for college adjustment, but the indirect effects (r = 0.393, *p* = 0.003) and the total effects (r = 0.491, *p* = 0.005) were significant. The indirect effects (r = −0.012, *p* = 0.158) were not significant for the sense of ego-identity on college adjustment, but the direct effects (r = 0.727, *p* = 0.004) and the total effects (r = 0.716, *p* = 0.005) were significant.

## 4. Discussion

This study identified the factors influencing college adjustment developed a model showing the direct and indirect effects between the relevant factors. Here, we intend to discuss the validity of the college adjustment structural model and the relationship between the factors influencing college adjustment based on the results of this study.

As a result of constructing the hypothetical model in this study, it was found that social support had a direct and positive effect on ego-identity, but negative child rearing attitude did not have a significant effect. Social support is important for a successful transition from adolescence to adulthood [34]. According to previous studies, social support from parents and other important people around students helps those students positively form self-confidence [35]. Thus, the more college students experience the social support they obtain from their surrounding interpersonal relationships, the more they can recognize themselves by integrating their ideals, behaviors, and social roles. As such, social support is important for college students to recognize themselves and prepare for a positive future. Thus, students should understand and actively support each other alongside the people around them such as family, friends, and professors.

On the other hand, based on the data of first-year college students in 2016 in a study on the Korean Children and Youth Panel, the children of ‘helicopter-type’ parents who are overprotective have high confidence for the future but poor goal orientation and intimacy [36]. In addition, the children of parents who show indifferent child rearing attitudes tend to avoid problems or decisions regarding their identity [37]. As such, in previous studies, child rearing attitudes were shown to have a significant effect on ego-identity [37], but this study produced different results. There are various types of child rearing attitudes, such as affectionate child rearing attitudes, autonomous child rearing attitudes, controlled child rearing attitudes, and rejective child rearing attitudes. However, in this study, since only rejective and overprotective child rearing attitudes were composed of sub-domains, it seems that the effects of parenting attitudes on self-identity were not sufficiently explained.

Negative child rearing attitude and social support negatively affected aggression. SNS addiction proneness had a positive effect on aggression. College students are under stress from various life problems, the more they perceive this burden, and the more they experience social exclusion, the more aggressive they become [38]. This is consistent with the results of the present study’s results showing that negative child rearing attitudes and social support affect aggression. When college students have unstable relationships with their parents, friends, and professors, the stress they experience while preparing for schoolwork and employment can express itself as an explosion. Therefore, it is important that parents, friends, and professors support college students to facilitate students’ psychological stability. Even if they have a negative impact on the individual, it is required to develop and apply programs that can overcome them. In addition, since SNSs offer exchanges in real time without the physical distance limitations of in-person human relationships, they can be helpful to form interpersonal relationships and communicate smoothly, but problems may arise due to their excessive use. As the use of SNS increases, withdrawal symptoms and addiction tendencies may occur, resulting in fatigue, decline in academic performance, gaps with reality, and difficulties in daily life [38].

In the same vein as SNS addiction proneness, as smartphones and the internet spread rapidly, resulting problems are beginning to emerge. Previous studies reported that mental-health problems such as fatigue, depression, lethargy, low self-esteem, anxiety, aggression, and suicidal thoughts are partially due to addiction to smartphones, the internet, and SNSs [38]. Thus, it is important to understand the proneness of college students to SNS addiction, and the previous results are consistent with the present results of this study showing that SNS addiction proneness and aggression are directly related. Therefore, it is necessary to educate college students about the positive and negative aspects of human relationships formed in SNS and strive to maintain positive interpersonal relationships. In addition, it is very important to develop coping strategies that can lower SNS addiction proneness and aggression and intervene accordingly.

The results of this study found that a negative child rearing attitude directly negatively affected SNS addiction proneness, but social support did not have a significant effect. According to a previous study that analyzed data from the 7th Korean Child and Adolescent Panel Survey in 2016, the higher the degree of dependence on smartphones was in the college student panel, the more severe the students’ parental overprotection (or the lower their parental supervision) [39]. Negative child rearing attitudes directly and indirectly worsened the degree of smartphone addiction among college students [40]. There are many related negative studies on smartphone addiction, but the standards for SNS addiction have not yet been established, so such an addiction cannot be clearly defined. However, many cases of dependence on smartphones lead to SNS addiction and smartphone addiction [41]. Based on previous studies and the results of this study, it can be seen that child rearing attitudes are closely related to SNS addiction. Thus, it is thought that one can prevent social media addiction by reducing negative child rearing attitudes, such as rejecting or overprotecting children, and strengthening positive child rearing attitudes through parental education.

On the other hand, in this study, social support did not have a significant effect on SNS addiction proneness. Previous studies have shown that social media addiction tends to increase when one feels psychological instability toward oneself, such as due to fear of alienation [42]. In addition, it was found that when feelings of attachment to others is high, students spend more time on SNSs, and when negative emotions arise, students also use SNSs [43]. On the other hand, for extroverts who have a large number of friends and pursue their senses, SNS addiction proneness was high [44]. In terms of relationships with others, the results of social media addiction proneness were very different depending on the student’s relationship with his or her friends. This suggests that for the social support variable, including friend support, opposite results may be produced depending on the study subject. Thus, a study to compare and analyze various subjects is needed in the future.

In the results of this study, social support indirectly had a positive effect on college adjustment, and ego-identity directly had a positive effect. Although social support did not directly significantly affect college adjustment, it indirectly influenced college adjustment through ego-identity. A direct comparison here is difficult since there are no prior studies in which variables were set equally between social support and college adjustment, like the path established in this study. However, based on the results of previous studies [45] showing that social support has an indirect effect on college adjustment through academic stress, it can be seen that social support is a variable that indirectly affects college adjustment. In the results of this study, social support did not directly affect college adjustment. However, the behavioral factor, which is an aspect of social support, increases ego-identity, which is an individual factor and has a positive influence on the behavioral factor (i.e., college adjustment).

In addition, we found that ego-identity has a direct and significant effect on college adjustment. In college, students explore their awareness of reality and begin to see themselves realistically. Ego-identity is formed along with self-reflection. Therefore, college students have their own subjectivity, accept and analyze themselves, and individually consider their goals and future. These factors are all important for college life and have a positive effect on college adjustments.

Based on these discussions, we will now suggest interventions and strategies to promote social support and ego-identity for college adjustments. To increase the social support of college students, program-based activities for friendship and smooth communication between seniors and juniors, friends, and professors are necessary. In addition, it is necessary to actively induce student participation and communication with professors through programs that allow students to adjust to college life and make future career choices. To facilitate the relationships between seniors, juniors, and friends, a mentoring program should be implemented to create and maintain intimate relationships, and efforts should be made to ensure that students are psychologically stable with continued interest and support from their parents. In addition, to improve the ego-identity of college students, it would be helpful to construct and provide various counseling programs that can enhance ego-identity in the long term, rather than using a one-time program.

Prior studies related to college adjustment focused on stress and coping, emphasized student interactions with the environment from a socio-cultural perspective, or focused on interpersonal relationships with early caregivers. However, this study is significant in that it constructed and analyzed a hypothetical model by explaining the college adjustment of college students based on Bandura’s social cognitive theory. College adjustment was identified by determining individual dispositions and various relationship patterns, and relevant data to explain college adjustments were presented, which could serve as the basis for future research. In addition, this study attempted a nursing-based interpretation of general college students’ adjustments to college by targeting a variety of departments, not only nursing. In terms of nursing practice, this study can provide a framework and be used as basic data when developing nursing intervention programs for college students to adjust to college. In addition, when engaging in nursing for college adjustment, this model could help enhance ego-identity and provide a theoretical basis for suggesting relevant and effective intervention directions for family, friends, and professors.

## 5. Conclusions

The structural model of college adjustment constructed in this study was shown to be suitable as a model to explain college adjustment. Social support and ego-identity were identified as important variables influencing college adjustment, and their explanatory power for college adjustment was 57.1%. Based on these variables, the present structural model is expected to contribute to mediation so that students can adapt positively to college life.

The following are possible avenues for future research. First, it is necessary to divide college adjustments into more detailed factors and further study various variables. Second, since the aspects of maternal rejection and overprotection among child rearing attitudes were used in the study, further studies that include the positive and negative parental child rearing attitudes of both parents are needed. Third, there are limitations in generalizing the present research results because the subjects were selected by convenience sampling. Therefore, it is necessary to repeat the present research while considering the distribution of each major targeting college students in various regions. Fourth, to increase college adjustment, research is required to develop and apply nursing education programs to increase ego-identity and maintain positive social supports for college students. Fifth, there is a need for practical measures that can be applied and operated by universities to increase social support and ego-identity, rather than focusing on improving knowledge or intellectual abilities in college education. Instead of emphasizing the interaction of students, it is to provide appropriate and various rewards (support for expenses used for exchange activities, evaluation of achievements by inducing interaction with students, etc.). In addition, specific measures for students’ ego-identity formation and college adjustment should be devised to guide students.

## Figures and Tables

**Table 1:** General characteristics of the participants.

Characteristics	Categories	N	%
Gender	Male	118	40.7
	Female	172	59.3
Grade	1st	97	33.4
	2nd	82	28.3
	3rd	63	21.7
	4th	48	16.6
Major	Humanities/Social science	44	15.2
	Education	28	9.7
	Engineering	61	21.0
	Natural science	43	14.8
	Health science	78	26.9
	Arts/physical	36	12.4
Religion	Christian	75	25.9
	Catholic	20	6.9
	Buddhist	24	8.3
	None	171	59.0
Father’s education	≤High school	104	35.9
	≥College/University	186	64.1
Mother’s education	≤High school	129	44.5
	≥College/University	161	55.5
Economic status	Low	54	18.6
	Middle	157	54.1
	High	79	27.3
Satisfaction with major	Very dissatisfied	9	3.1
	Dissatisfied	27	9.3
	Average	109	37.6
	Satisfied	97	33.4
	Very satisfied	48	16.6
Use of SNS	Yes	270	93.1
	No	20	6.9
Spent time on SNS in day (minutes)	<59	42	14.5
	60–119	73	25.2
	120–179	92	31.7
	180–239	39	13.4
	≥240	44	15.2
Number of SNS connections per week	≥7	213	73.4
	5–6	28	9.7
	3–4	19	6.6
	≤2	30	10.4
Maintain relationship with SNS	Yes	159	54.8
	No	131	45.2
Relationships maintained only by SNS	None	94	32.4
	Less than half	106	36.6
	Half	54	18.6
	More than half	20	6.9
	Most	16	5.5
Problems between friends due to SNS	Yes	41	14.1
	No	249	85.9

**Table 2:** Descriptive statistics for the measured variables.

Variable (Item)	Range	Average		Skewness	Kurtosis	AVE	CR
		M	SD				
Negative child rearing attitude (15)						0.39	0.56
Maternal rejection (7)	1–4	1.39	0.50	1.78	3.66		
Maternal overprotection (8)	1–4	1.88	0.60	0.90	0.24		
Social support (12)						0.38	0.65
Family support (4)	1–5	4.17	0.86	−1.06	0.82		
Friends support (4)	1–5	4.17	0.82	−0.97	0.88		
Significant other support (4)	1–5	3.80	1.16	−0.80	−0.26		
Ego-identity (50)						0.49	0.82
Initiative (10)	1–5	3.70	0.53	−0.03	−0.08		
Self-receptiveness (10)	1–5	3.75	0.74	−0.46	−0.20		
Confirmation for the future (10)	1–5	3.60	0.70	−0.10	−0.62		
Goal orientation (10)	1–5	3.25	0.67	0.14	−0.23		
Intimacy (10)	1–5	3.38	0.71	0.08	−0.58		
SNS addiction proneness (24)						0.63	0.87
Disturbance of adaptive life and control (7)	1–4	1.81	0.62	0.35	−0.63		
Preoccupation and tolerance (7)	1–4	1.96	0.60	0.38	−0.42		
Avoidance of negative emotions (5)	1–4	1.82	0.66	0.64	−0.02		
Virtual life orientation and withdrawal (5)	1–4	1.73	0.61	0.73	0.19		
Aggression (27)						0.57	0.84
Physical aggression (9)	1–5	1.90	0.67	1.34	1.98		
Verbal aggression (5)	1–5	2.27	0.75	0.79	0.48		
Anger (5)	1–5	2.28	0.73	0.57	0.03		
Hostility (8)	1–5	2.01	0.74	0.96	0.92		
College adjustment (12)						0.63	0.83
Academic activities (4)	1–5	3.59	0.74	−0.14	−0.33		
Career preparation (4)	1–5	2.77	0.69	−0.01	−0.09		
Personal psychology (4)	1–5	3.59	0.75	−0.22	0.01		

M = mean; SD = standard deviation; AVE = average variance explained; CR = construct reliability.

**Table 3:** Standardized Estimates, Critical Ratio (C.R.), Squared Multiple Correlations (SMC), Standardized Direct, Indirect, and Total Effects for the Modified Model.

Endogenous Variables	Exogenous Variables	SE	C.R (*p*)	SMC	Standardized Direct Effect (*p*)	Standardized Indirect Effect (*p*)	Standardized Total Effect (*p*)
Ego-identity	Negative child rearing attitude	−0.03	−0.09 (0.194)	0.357	−0.088 (0.348)		−0.088 (0.348)
	Social support	0.20	0.57 (<0.001)		0.567 (0.003)		0.567 (0.003)
SNS addiction proneness	Negative child rearing attitude	0.52	0.33 (<0.001)	0.127	0.331 (0.005)		0.331 (0.005)
	Social support	−0.10	−0.07 (0.323)		−0.070 (0.397)		−0.070 (0.397)
Aggression	Negative child rearing attitude	0.52	0.33 (<0.001)	0.294	0.328 (0.005)	0.069 (0.005)	0.396 (0.003)
	Social support	−0.23	−0.16 (0.037)		−0.161 (0.048)	−0.067 (0.116)	−0.228 (0.005)
	Ego-identity	−0.39	−0.10 (0.222)		−0.096 (0.190)		−0.096 (0.190)
	SNS Addiction proneness	0.19	0.18 (0.004)		0.182 (0.017)		0.182 (0.017)
College adjustment	Negative child rearing attitude	0.07	0.19 (0.051)	0.571	0.189 (0.089)	−0.056 (0.495)	0.133 (0.129)
	Social support	0.04	0.10 (0.245)		0.098 (0.385)	0.393 (0.003)	0.491 (0.005)
	Ego-identity	0.73	0.73 (<0.001)		0.727 (0.004)	−0.012 (0.158)	0.716 (0.005)
	SNS addiction proneness	−0.03	−0.12 (0.054)		−0.121 (0.114)	0.022 (0.091)	−0.099 (0.174)
	Aggression	0.03	0.12 (0.083)		0.120 (0.162)		0.120 (0.162)

SE = standard error; CR = critical ratio; SMC = squared multiple correlation.
